# Climate change drives migratory range shift via individual plasticity in shearwaters

**DOI:** 10.1073/pnas.2312438121

**Published:** 2024-01-29

**Authors:** Patrick J. Lewin, Joe Wynn, José Manuel Arcos, Rhiannon E. Austin, Josephine Blagrove, Sarah Bond, Gemma Carrasco, Karine Delord, Lewis Fisher-Reeves, David García, Natasha Gillies, Tim Guilford, Isobel Hawkins, Paris Jaggers, Christian Kirk, Maite Louzao, Lou Maurice, Miguel McMinn, Thierry Micol, Joe Morford, Greg Morgan, Jason Moss, Elisa Miquel Riera, Ana Rodriguez, Katrina Siddiqi-Davies, Henri Weimerskirch, Russell B. Wynn, Oliver Padget

**Affiliations:** ^a^Department of Biology, University of Oxford, Oxford OX1 3SZ, United Kingdom; ^b^Institut für Vogelforschung “Vogelwarte Helgoland”, Wilhelmshaven 26386, Germany; ^c^Programa Marino, Sociedad Española de Ornitología/BirdLife, Delegació de Catalunya, Barcelona 08026, Spain; ^d^National Oceanography Centre–Southampton, Southampton SO14 3ZH, United Kingdom; ^e^Earth Ocean and Ecological Sciences, School of Environmental Sciences, University of Liverpool, Liverpool L69 3GP, United Kingdom; ^f^School of Ocean Sciences, College of Science and Engineering, Bangor University, Menai Bridge LL59 5AB, United Kingdom; ^g^Iniciativa de Recerca de la Biodiversitat de les Illes, Alaior, Balearic Islands 07730, Spain; ^h^Centre d’Etudes Biologiques de Chizé, Laboratoire des Sciences de l'Environnement Marin, UMR 7372, Centre National de la Recherche Scientifique, Villiers en Bois 79360, France; ^i^AZTI, Marine Research, Basque Research and Technology Alliance, Pasaia 20110, Spain; ^j^British Geological Survey, Wallingford OX10 8ED, United Kingdom; ^k^Grupo Biogeografía, geodinámica y sedimentación del Mediterráneo occidental, Ciències i Tecnologies Mediambientals, Universitat de les Illes Balears, Palma, Balearic Islands E07122, Spain; ^l^Ligue pour la Protection des Oiseaux, BirdLife International Partner in France, Rochefort Cedex 17305, France; ^m^Royal Society for the Protection of Birds, Ramsey Island, St. Davids, Pembrokeshire SA62 6PY, United Kingdom

**Keywords:** climate change, plasticity, migration, range shift, spatial cognition

## Abstract

How individual animals respond to climate change is key to whether populations will persist or go extinct. Yet, few studies investigate how changes in individual behavior underpin these population-level phenomena. Shifts in the distributions of migratory animals can occur through adaptation in migratory behaviors, but there is little understanding of how selection and plasticity contribute to population range shift. Here, we use long-term geolocator tracking of Balearic shearwaters (*Puffinus mauretanicus*) to investigate how year-to-year changes in individual birds’ migrations underpin a range shift in the post-breeding migration. We demonstrate a northward shift in the post-breeding range and show that this is brought about by individual plasticity in migratory destination, with individuals migrating further north in response to changes in sea-surface temperature. Furthermore, we find that when individuals migrate further, they return faster, perhaps minimizing delays in return to the breeding area. Birds apparently judge the increased distance that they will need to migrate via memory of the migration route, suggesting that spatial cognitive mechanisms may contribute to this plasticity and the resulting range shift. Our study exemplifies the role that individual behavior plays in populations’ responses to environmental change and highlights some of the behavioral mechanisms that might be key to understanding and predicting species persistence in response to climate change.

## Significance

We repeatedly tracked Balearic shearwaters, allowing us to investigate how changes in migratory behavior generate range-shift under climate change. Range-shifts are often viewed only as population-level phenomena but we show that, in this long-lived seabird, individuals flexibly respond to increasing temperatures throughout their lives by migrating further north. We link this plasticity to changes in migratory behavior and associated spatial cognition. In particular, shearwaters appear to regulate the speed of return migration by estimating the distance of the migratory route. Nonetheless, this increased speed is insufficient to avoid delayed return phenology. These findings contribute to our understanding of how individual flexibility in migratory behaviors and spatial cognition might allow animals to track shifting resources under climate change.

Understanding the biological impact of climate change is contingent upon understanding species’ responses at every level: from population-level phenomena, through the processes of selection and plasticity, to the behavioral and cognitive mechanisms that underpin these changes. Adaptation in response to environmental change might occur within or between individuals (1, 2); adaptive genotypic evolution occurs through selection between individuals with different strategies and developmental plasticity [sensu Piersma & Drent (3)] involves fixed periods of adaptation within individuals, producing adults with fixed strategies and generational changes at the population level. In contrast, within-individual phenotypic flexibility (3) allows reversible adaptation within individual lifetimes. Range shift, usually viewed as a population-level result of climate change (4–6), can also be understood as the result of adaptation in spatial behaviors, including migration (7–9). In previous empirical work, range shift has mostly been found to result from turnover of individuals with fixed adult migratory routes and destinations, with one recent exception (8–10). However, the potential role of phenotypic flexibility in contributing to migratory range shift remains little investigated, as do the potential navigational mechanisms that might underlie flexibility in migratory behaviors.

Balearic shearwaters are a Critically Endangered procellariiform seabird which breed exclusively in the Balearic Islands from March to early July, before the majority of the population migrate out of the Mediterranean Sea, into the North Atlantic, via the Strait of Gibraltar between April and September (11–14). Individuals spend variable amounts of time along the western coasts of Europe and Morocco before returning between May and January for an extended pre-breeding period in the western Mediterranean (11, 12). Most individuals migrate either to the western and southwestern coasts of Iberia, or to a spatially separated area further north (11, 15), in the Bay of Biscay and around Brittany. Analysis of sightings data has suggested a rapid northward shift in the species’ Atlantic range in the mid-1990s, associated with rising Sea-Surface Temperatures (SST) and shifting clupeid fish stocks (16, 17). However, this trend might be subject to bias, resulting from increased observer interest (18), due to the species’ changing taxonomic status (19–22), or could result from other temporally correlated variables (18). Nonetheless, if there is an ongoing range shift in the species’ post-breeding distribution, Balearic shearwaters might be particularly likely to exhibit flexibility in migratory destination. Like other procellariiforms, they can be extremely long-lived (23, 24), are capable of long foraging trips during the breeding season (25, 26), and are generally dependent on patchy and ephemeral resources which might shift rapidly with climate change (27, 28). Furthermore, shearwaters have become a model system for the study of avian spatial cognition in the wild (26, 29–35). As a result, Balearic shearwaters provide an excellent opportunity to explore the processes generating range shift in migratory taxa and investigate the underlying behavioral and spatial cognitive mechanisms.

**Fig. 1.**
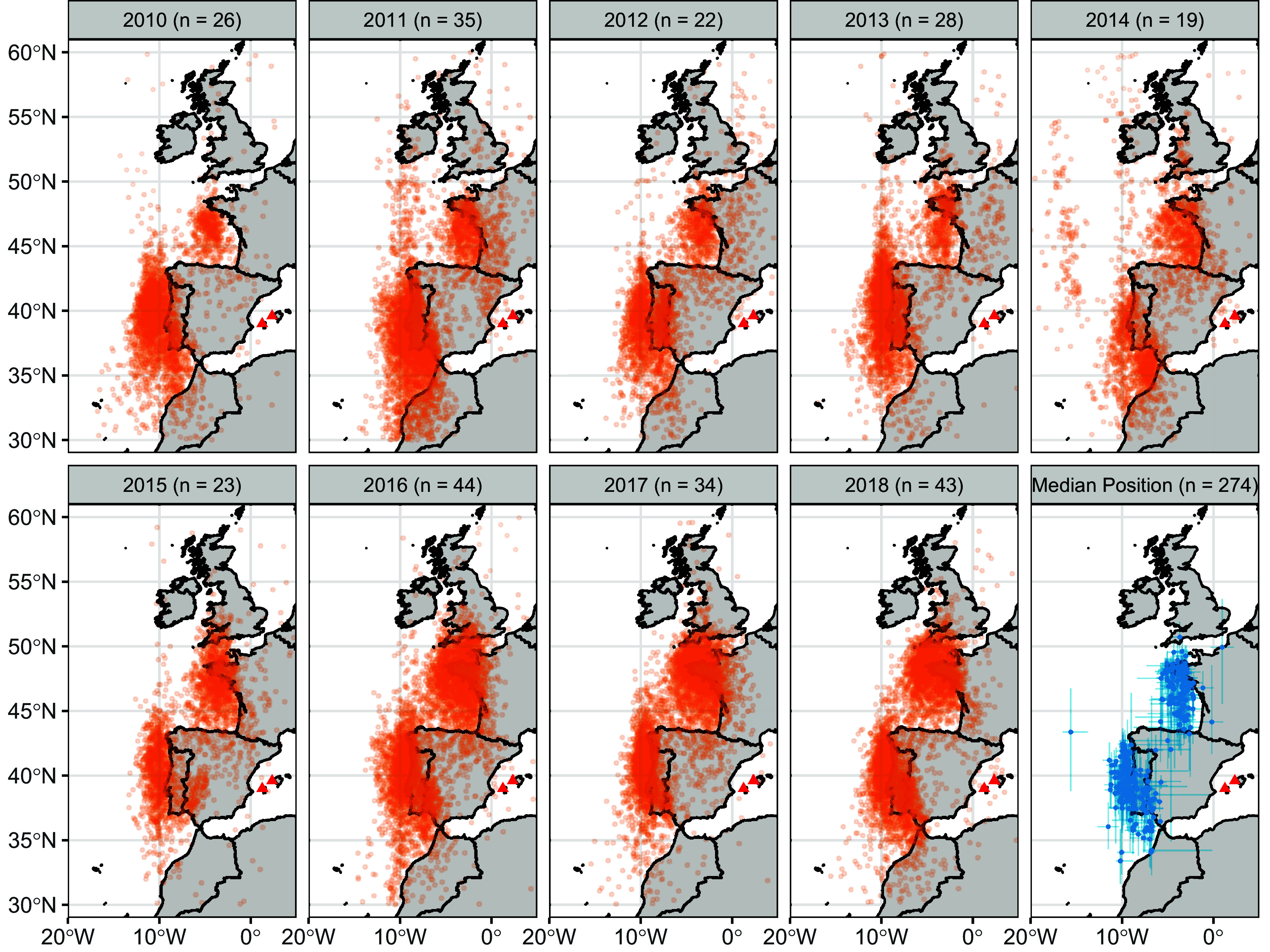
The post-breeding migration of the Balearic shearwater. Maps show all location estimates for all birds in each year which were identified as being part of a migration. The final panel shows the median and interquartile range of latitude and longitude during each separate migration. Breeding locations are shown with red triangles; the Ibizan colonies of Sa Conillera and Es Bosc are to the west, the Mallorcan colonies of Sa Cella and Sa Dragonera are to the East.

Here, we used light-level geolocators (274 migrations by 145 individuals) to track Balearic shearwaters breeding on the Balearic Islands of Ibiza and Mallorca over the course of a decade and investigate their response to climate change (Fig. 1). We demonstrate a northward range shift in the species’ post-breeding distribution, facilitated at least in part by phenotypic flexibility in migratory destination, and show that shearwaters compensate for the resulting increase in migratory distance by travelling faster on return migration. Differences in return distance appear to be anticipated from the start of return migration, based on route learning. Despite compensatory increases in speed, when shearwaters must return from further north, they return to the breeding area later.

## Results

### Northward Range Shift and Phenotypic Flexibility in Migratory Destination.

**Fig. 2.**
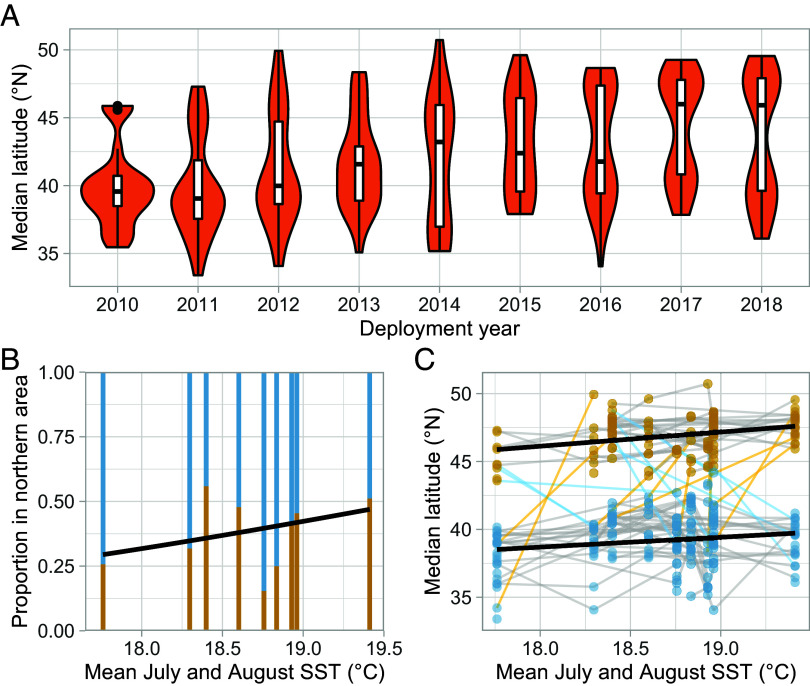
The northward range shift in Balearic shearwaters summarized through time and with respect to SST. (*A*) The distribution of median latitudes over each deployment year of the study. (*B*) The proportion of birds migrating to the northern or southern area throughout the study, with respect to SST. The northern area (Biscay) is represented in gold. The fitted line is a logit-linked generalized linear regression between this proportion and SST in each year. (*C*) The relationship between average SST and median migratory latitude. The median latitude observed during each separate migration is represented as a single point; those identified as being in the northern area are gold and those in the southern area are blue. All observations relating to a single individual are joined by lines; gold lines indicate cases in which an individual has switched from the southern to the northern area between years and blue lines cases in which individuals have switched from the northern to southern area. The shift in migratory latitudes in each area is shown using a simple least-squares regression.

We calculated the median latitude of each bird during each migration and used mixture models to identify whether the bird migrated to the northern (Biscay) or southern (Iberia) area (see *Materials and Methods* and Fig. 5). This allowed us to differentiate year-on-year movements within and between the two discrete areas. Using a linear regression, with median latitude from each bird’s first recorded migration as the response, we found a significant northwards shift of 0.2317° (±0.07339°) per year (F_4,93_ = 108.5515, *P* < 1 × 10^−15^; Fig. 2*A*). To test whether this change was in response to climate, we calculated the mean July and August SST across the post-breeding region (12°W to 1°W and 38°N to 51°N; see *Materials and Methods*) and compared two non-nested models: median latitude by year and median latitude by SST. The model using year as the predictor fitted substantially worse than a model using SST (AICc = 393.93 and 383.08, respectively, ΔAICc = −10.85), indicating that climate-induced changes best explain this range shift.

We aimed to distinguish whether range shift resulted from phenotypic flexibility in migratory latitude within individuals or turnover of individuals with fixed migratory latitudes. We used van de Pol & Wright’s method (36) to calculate the within- and between-individual slopes (*Materials and Methods*). Significance is reported from likelihood ratio tests on nested full and null models for each predictor. Effect sizes are reported ± their bootstrapped 95% CI and the significance of differences between effect sizes for within- and between-individual components are assessed using the bootstrapped CI of these differences (*Materials and Methods*). Full model structures are reported in the *Materials and Methods* and full model results in *SI Appendix*, Table S1.

First, we found that the northward range shift was at least partially explained by within-individual phenotypic flexibility in migratory latitude (within-individual slope: χ^2^ 1 = 4.063, *P* = 0.044; between-individual slope: χ^2^_1_ = 12.07, *P* = 0.001; Fig. 2*C*). The between-individual slope was larger (1.421 ± 0.80° latitude per °C) than the within-individual slope (0.662 ± 0.65° latitude per °C), but there was no significant difference between these effect sizes (95% CI of difference: −0.262, 1.763) suggesting that phenotypic flexibility in migratory latitude, but not turnover of individuals, has a detectable contribution to the population-level shift.

We also tested for changes in the probability that birds migrated to either the northern or southern area, using a binomial generalized linear mixed effects model with a logit link function. We included island of origin as a fixed effect in the model but this term had no significant effect, indicating that birds from different islands migrated to each area in similar proportions (χ^2^_1_ = 1.246, *P* = 0.264). In contrast to changes in latitude within each area, we found that there was no significant within-individual effect of temperature on which area birds migrated to (χ^2^_1_ = 0.0001, *P* = 0.988). We found that, between individuals, the log odds of birds migrating to the northern area increased by 1.79 for every increase of 1 °C (χ^2^_1_ = 4.560, *P* = 0.03274; Fig. 2*B*). Despite the significance indicated by the likelihood ratio test, the bootstrapped CI of i) the between-individual effect (−0.234, 3.893) and the ii) the difference between the within- and between-individual effect sizes (−0.563, 0.535) overlapped with zero. Consequently, this result should be interpreted as inconclusive.

### Differences in Return Speed Based on Route Memory.

If shearwaters migrate further north but their breeding sites remain fixed (37, 38), they must either migrate faster or spend longer migrating. We investigated whether shearwaters’ return speed was related to the latitude they migrated to and whether any change was explained by within-individual phenotypic flexibility in migration speed or turnover of individuals with fixed speeds. We split each migration into the outbound and return migration either side of its maximum latitude (*Materials and Methods*) and calculated the mean daily speed over each section. To control for physically constrained individual differences in speed and the possibility that faster birds inherently reach higher latitudes, we included the mean outbound speed in our model, so that residual variation probably reflected decisions made by the bird. We also included whether birds migrated to the northern or southern area as an additive factor in the model, to account for the confound that birds migrating to the northern area necessarily cross different habitats.

**Fig. 3.**
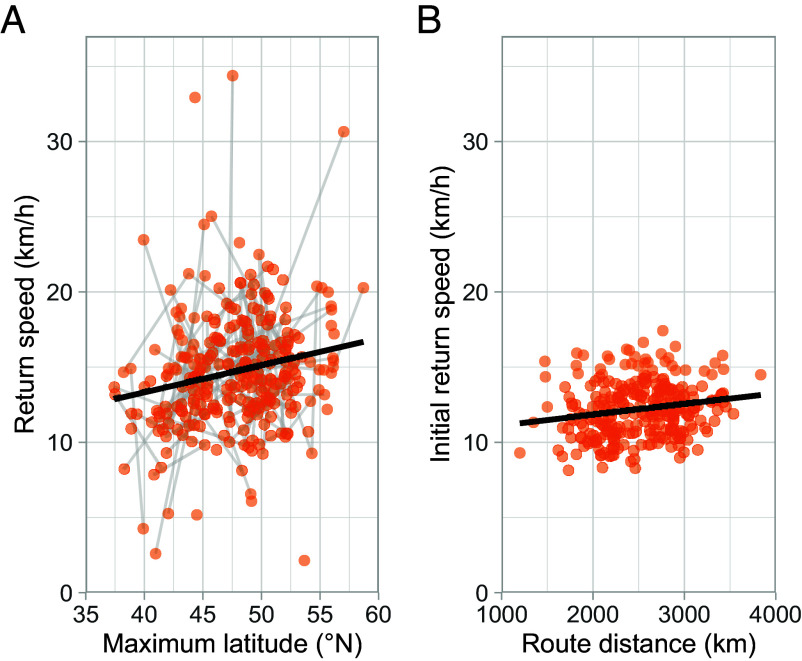
Average travel speed during return migration. Each migration was split into outbound and return sections at the apex. The average daily speed over the entire return section and over the initial 25% was calculated. These speeds on each migration are represented as single points. (*A*) Overall return speed is regressed against maximum latitude for each migration and points are joined by lines for all observations of a single individual. (*B*) Average speed in the initial 25% of return migration against route distance, the best-fitting distance predictor.

Return speed was not significantly related to outbound speed (χ^2^_1_ = 2.658, *P* = 0.103), suggesting that outbound and return speed are determined independently and are not simply under a joint biomechanical constraint. We found significant increases in return speed with maximum latitude; shearwaters which fly further north travel faster on return migration. The between- and within-individual slopes were almost identical (0.227 ± 0.15 km/h per 1° maximum latitude, χ^2^_1_ = 8.517, *P* = 0.004 and 0.207 ± 0.20 km/h per 1°, χ^2^_1_ = 4.055, *P* = 0.044, respectively; Fig. 3) and there was no significant difference between these effect sizes (95% CI: −0.226, 0.257), suggesting that changes in speed in response to more northerly migratory destinations can be accounted for by individual flexibility alone.

Increases in migration speed could be facilitated by two mechanisms, with different cognitive implications: birds might estimate the distance of their return migration and increase their speed from the very start of the return, or they might only speed up reactively if they perceive that they have failed to reach their goal after a certain time. We calculated speed in the first 25% of return migration to test whether birds were estimating return distance and planning ahead by adjusting initial return speed accordingly.

We also investigated what kind of spatial representation might underlie estimates of the distance home from the start of return migration. Exploiting the fact that shearwaters avoid flying far over land, we constructed two models based on different potential spatial representations. The first used the beeline distance directly back to the colony (regardless of any intervening land) which could be based on a grid map representation (26). The second used the distance along the shortest sea route, which could be based on route memory of previous migrations. The variation in these distances is large and not perfectly correlated, and we used non-nested model comparisons to investigate which distance better explained changes in speed with migratory latitude. We found that the model using the beeline distance fitted worse (AICc = 1,117.64) than the model using route distance (AICc = 1,112.64, ΔAICc = −5.00). Within the better fitting model, initial return speed increased by 0.0713 km/h for every increase in distance of 100 km (F_1,272_ = 8.931, *P* = 0.00306; Fig. 3), suggesting that the shearwaters use an estimate of the route distance to adjust their speed from the start of return migration. Given this effect size, the furthest birds return 2 d earlier than they would have if they flew at the same speed as the closest birds, on average. To rule out the possibility that this result is driven by the inherent relationship between latitudinal error in geolocation and the estimated speed, we also fitted a model predicting initial return speed from maximum latitude (AICc = 1,117.31). The model fit was marginally better than the beeline model (ΔAICc = −0.33) and substantially worse than the route distance model (ΔAICc = 4.67). This strongly suggests that this result is not an artifact of geolocation error. Shearwaters migrating from different latitudes must encounter different environmental conditions at the start of their return migrations, creating a potential confound between maximum latitude and environmental correlates which influence migration speed. To ensure that this was not driving the relationship between maximum latitude and return speed, we calculated the average speed for each track over a fixed latitudinal band (35°N to 45°N) through which all shearwaters must migrate, passing over the same area and habitat. The positive effect of maximum latitude on return speed was still maintained over this fixed area (0.218 ± 0.17 km/h per °N, χ^2^_1_ = 6.357, *P* = 0.012), ruling out the effect of differences in habitat. Hence, birds appear to learn about their route and use this memorized distance to anticipate how long their return journey will be.

### Phenological Shifts.

**Fig. 4.**
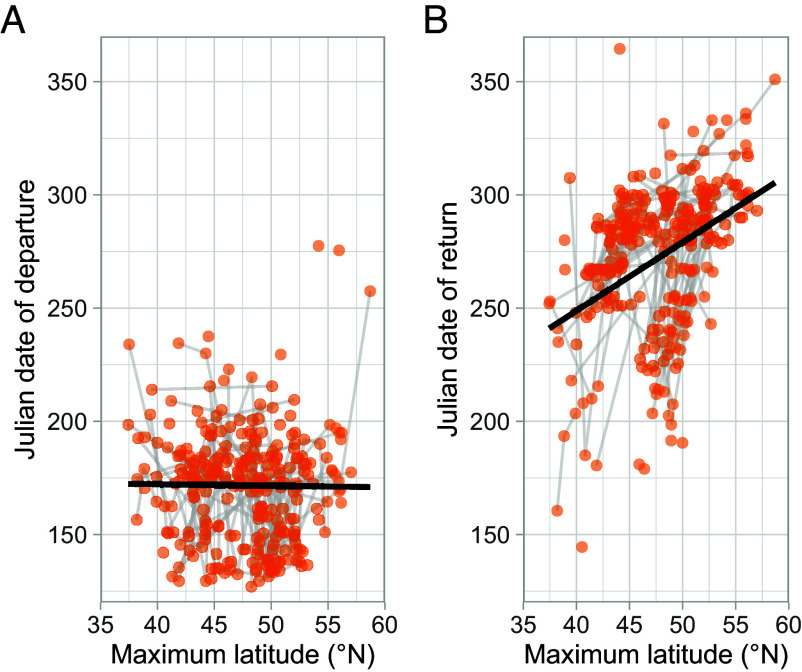
The effect of migratory latitude on phenology. Migration was identified as the longest period outside the Mediterranean Sea. The start of migration was identified as the last time the shearwater left the Mediterranean Sea before this period and the end of migration as the first time it returned to the Mediterranean Sea. The dates of these phenological markers are represented as single points for each migration, and the points belonging to single individuals are joined by the gray lines. The fitted lines are least-squares regressions between phenology and maximum migratory latitude. (*A*) Date of departure from the Mediterranean Sea against maximum latitude. (*B*) Date of return to the Mediterranean breeding area against maximum latitude.

Despite this compensatory increase in speed, migratory phenology might still be affected by the range shift or associated environmental changes. This could be because breeding phenology might advance under climate change (4, 39), because higher temperatures in post-breeding areas might prolong their suitability or because the increase in speed might not be sufficient to compensate for the increased migratory distance. There was no significant between- or within-individual relationship between maximum latitude and when shearwaters first left the Mediterranean Sea (χ^2^ 1 = 1.878, *P* = 0.171 and χ^2^_1_ = 0.259, *P* = 0.611, respectively; Fig. 4), and there was no difference between breeding islands (χ^2^_1_ = 0.180, *P* = 0.672). However, shearwaters migrating to the southern area left the Mediterranean Sea 11.4 ± 7.3 d later, on average (χ^2^_1_ = 6.497, *P* = 0.002).

The date of return to the Mediterranean Sea was, however, significantly predicted by island of origin, whether birds migrated to the northern or southern area and maximum latitude. Mallorcan shearwaters returned to the Mediterranean Sea an average of 9.8 ± 6.787 d earlier than Ibizan shearwaters (χ^2^_1_ = 8.797, *P* = 0.003) and those in the southern area returned an average of 38.5 ± 8.0 d later (χ^2^_1_ = 80.549, *P* < 1 × 10^−15^) than those from the north. Furthermore, within each area, shearwater return phenology was delayed as maximum latitude increased, with almost identical between- and within-individual slopes (5.4 ± 1.0 d per 1°, χ^2^_1_ = 81.778, *P* < 1 × 10^−15^; 4.4 ± 1.4 d per 1°, χ^2^_1_ = 42.532, *P* < 1 × 10^−10^; Fig. 4), and no significant difference in effect size (95% CI: −0.637, 2.396).

## Discussion

The post-breeding migration of Balearic shearwaters has shifted northward over the course of a decade, under a changing climate. This shift appears to be facilitated by phenotypic flexibility in migratory destination and, in turn, increasing migration speeds based on memory of migratory route length. Whilst we use SST early in the migration season as a proxy for climate, it is unlikely that shearwaters are responding to temperature directly. Instead, Balearic shearwater distributions are probably determined by a number of environmental factors (40) and shearwaters may be responding more directly to food availability, as suggested by Luczak et al. (17). We also acknowledge that analyses using geolocator data, especially those relating location estimates to environmental conditions, are susceptible to artifacts caused by the effects of those environmental conditions on light levels (41). However, the observed range shift in this study is consistent with studies using other methods (16, 42) and we consider a consistent bias toward more northerly error in geolocation with increasing temperature to be unlikely. We, therefore, suggest that although error in geolocation is necessarily high, it is likely to be unbiased in this study.

The range shift we detect at the population level is underpinned by two migratory changes. First, shearwaters are increasingly likely to migrate to the northernmost of two spatially separated areas and, second, the median latitudes reached by individuals migrating to each of these areas are moving north. The latter of these is consistent with phenotypic flexibility [sensu Piersma & Drent (3)] in migratory destination; individual shearwaters alter their migratory destination as temperatures change. This is largely in contrast to previous investigations which have identified turnover of individuals with fixed migratory strategies as an adaptive process underlying range shift (8–10). Phenotypic flexibility could allow individuals to track shifting resources under climate change and so remain in a constant thermal niche, despite changing conditions. This individual-level response might contribute to the population persistence crucial to the conservation of this Critically Endangered species. This interpretation is supported by apparently constant breeding success and survival of Balearic shearwaters throughout this study, despite the changing environmental conditions and individual migratory behavior (*SI Appendix*).

The mechanisms that underpin such plasticity are poorly understood. We find evidence that spatial cognition might influence the capacity for population-level range shift by shaping individual changes in behavior in response to the environment; when migrating further north, shearwaters use memory of their migratory route distance to inform return migratory speed. This increase in speed has the potential to compensate for increased migration distance whilst phenology is held constant. Previous studies in the closely related Manx shearwater have shown that irrespective of intervening geographic obstacles birds tend to time long-distance movements based on the beeline distance to the colony, implying the use of a large-scale grid map (26). The use of route distance in Balearic shearwaters might instead reflect a cognitively simpler mechanism, where birds remember the length of return migration from previous years or from their outbound journey.

The second form of range shift is a change in the proportion of birds migrating to each area. This could be explained by turnover of individuals in the study population with changing temperature, as each individual consistently returns to one area every year [consistent with Meier (43)], although the results were equivocal. This result would be consistent with the few previous investigations of the mechanism of migratory range shift (8, 10) and might be the result of developmental plasticity or selection. Rates of turnover in the study population were high (*SI Appendix*, Fig. S3) but the constraints imposed on sampling by the inaccessibility of nest sites, along with inability to track juvenile and immature shearwaters before their first breeding attempts, makes it hard to distinguish selection from developmental plasticity because a) we cannot definitively detect mortality and b) we cannot currently investigate when or how migration preferences might develop with age. Remote-download tracking Balearic shearwaters’ first migrations showed that birds appear to select areas with lower SST and higher marine productivity (44) but these were incomplete migratory journeys and repeated tracking of entire migrations by young shearwaters would give important insight into the processes by which fixed migratory areas develop at the individual level and change through time at the population level.

Climatic changes frequently alter migratory phenology (4, 45–47) and Conklin et al. (48) showed for the first time that such changes could be explained entirely by phenotypic flexibility. Here, we also report a phenological change explained entirely by within-individual changes, but rather than an advancing departure, we see that birds are returning to the Mediterranean Sea later as a result of longer migrations, despite increased return speeds. This might be interpreted in several ways. Higher temperatures might keep northern waters suitable for longer into the post-breeding period, allowing shearwaters to prolong their time there before return migration. Alternatively, it might be that the delay is simply a result of having to migrate further. In this case, the delay might be costly, reducing the amount of time shearwaters have to regain condition after migration, or coordinate with a mate at the colony before breeding, which may influence breeding success (11). Furthermore, breeding phenology is generally expected to advance under climate change, in conflict with a delayed return from migration, and lack of advancement appears to be associated with population decline in birds (49). Since Balearic shearwaters are Critically Endangered, future research should aim to investigate the effects of climate change on the pre-breeding period and their link to breeding success and survival.

Most previous studies have considered climate-driven range shifts only at the population-level (4–6) so have not investigated the underlying adaptive processes in spatial behavior. Where studies have investigated these underlying processes, there has mostly been evidence for generational changes, indicative of selection or developmental plasticity (8–10). Our finding that a climate-driven range shift involves phenotypic flexibility in migratory destination demonstrates that life-long flexibility may play a role in some species and highlights the importance of considering range shifts not just as population-level phenomena but as the result of adaptation in spatial behavior. We clarify how this response is influenced by behavioral flexibility, itself underpinned by memory of migration routes, emphasizing the importance of spatial cognitive mechanisms in mediating population-level range shift. Nonetheless, the considerable delays to return phenology associated with northward range shift indicate that such flexibility could be insufficient to keep pace with climate change, for a species facing global extinction.

## Materials and Methods

### Fieldwork and Data Collection.

This study took place across four colonies of the Balearic shearwater: Sa Cella (39°36’N, 2°21’E) and Sa Dragonera (39°35’N, 2°19’E) on Mallorca, and Es Bosc (38°58’N, 1°13’E) and Sa Conillera (38°59’N, 1°13’E) on Ibiza, under permits issued by the Government of the Balearic Islands (CAP03/2011, CAP31/2011, CEP04/2012, CEP26/2012, ENB347/12, CEP03/2013, CEP09/2013, CEP15/2014, CEP19/2014, CEP/2015, CEP09/2015, CEP/2016, CEP18/2016, SEN224/17, ANE23/2017, ANE16/2018, CEP21/2018) and permission from the Oxford University Animal Welfare and Ethical Review Board. Geolocators were deployed on breeding birds between 2010 and 2018, using BAS MK15, MK18, and MK19, BioTrack MK3005, MK3006, and Migrate Tech C250, C330, and C65 loggers attached to a plastic leg ring, as per Guilford et al. (11), or directly to the bird’s permanent metal ring.

As a result of the inaccessibility of Balearic shearwater nesting sites, targeting of individual birds for geolocator deployments and retrievals was determined mostly by logistical constraints; nest sites were identified at the start of the study and returned to in future years, if access was still possible.

Monthly averaged SST data were accessed from the Copernicus ERA5 dataset (URL: https://cds.climate.copernicus.eu/). There is a consistent latitudinal SST gradient in the eastern North Atlantic Ocean during the post-breeding migration period. Consequently, as average temperatures increase, the latitudes at which a given temperature occurs shift northward. As a result, average temperature values over a fixed area during a fixed period in different years can act as a proxy for the latitudinal shift of climatic conditions. We defined a fixed area covering the core of the post-breeding distribution of Balearic shearwaters (12°W to 1°W and 38°N to 51°N) and calculated the mean July and August SST in each year, corresponding to when most Balearic shearwaters arrive in the Atlantic. This allowed us to set up the simple and testable prediction that as mean SST increases, shearwaters should shift northward if there is a climate-driven range shift.

### Geolocator Processing and Identification of Migration.

Geographic positions were estimated using the Geolight package (50) in R version 3.4.3 (51), with a twilight threshold of 10 lx and an elevation angle of −4.5°. This elevation angle was chosen based on visual assessment of the location of breeding season fixes in the Western Mediterranean Sea in a random subset of 40 tracks. Further processing followed Austin et al. (52), removing unreliable fixes around the equinoxes and those with unrealistic daily average speeds (>55 km/h). Unrealistic location estimates >22°W, >42°E, <30°N or >60°N were also removed. Tracks with fewer than 100 fixes or gaps of over 2 mo within 8 mo of tag deployment were excluded.

To isolate migratory behavior, we specified the date each bird left the Mediterranean Sea (via the Straits of Gibraltar) and the date at which it returned using a rolling mean longitude and latitude over a window of three fixes and a buffer area across mainland Europe (*SI Appendix*). When the rolling mean positions fell to the west of the buffer, they were identified as being in the Atlantic Ocean, on migration. When they fell east of the buffer, they were identified as being in the Mediterranean Sea. Those falling within the buffer were considered ambiguous and assigned the same value as the preceding location estimate, such that a change to or from migration was only identified when the rolling mean position fell unambiguously to one side of the buffer. The buffer is, at minimum, 170 km across, east to west, and 555 km across, north to south. Error in light-level geolocation is often high (53–57), especially in the latitudinal component (53). Given that Balearic shearwaters in the post-breeding migration are likely subject to only one of the three most significant causes of geolocator error in terrestrial settings [weather, topographical features, and vegetation, (54)], it seems reasonable to assume that error in our location estimates, especially after filtering, is likely to be at the lower end of these published estimates. It may be similar to data from Scopoli’s shearwaters (*Calonectris diomedia*) in the Mediterranean Sea, in which similar methods yielded mean estimates of 139 ± 127 km absolute error relative to co-deployed GPS (55). In addition, the use of a rolling mean longitude and latitude should further reduce error. We therefore believe that the chosen buffer is sufficient to rule out the vast majority of erroneously identified crossings.

Where a shearwater crossed into the Atlantic Ocean and back more than once in a single year, the longest period in the Atlantic Ocean was identified as the core period of migration and used for analyses of migratory behavior. The midpoint in time between the fixes either side of the buffer was used to represent the date of crossing. This allowed us to estimate the date of return even if it occurred during a gap between fixes of more than 1 d. Seven tracks did not include any migration fixes by this measure and a further two tracks ended before return to the Mediterranean Sea, leaving 274 tracks by 145 individuals for analysis, including 127 tracks from Mallorca and 147 from Ibiza.

We aimed to split each migration into outbound and return sections. Balearic shearwaters show great variation in the extent of migration and many individuals do not spend a prolonged period in a single location during the post-breeding season. As a result, we could not use clear differences in the rate of travel to identify the end of outbound and the start of return migration. Instead, we used the apex of each migration. We defined the apex as the fix closest to the 98th quantile of latitude (to exclude extreme outliers most likely to be the result geolocation error). All fixes before this were identified as part of the outbound migration and all fixes afterward as the return.

### Statistical Analysis.

Analysis was conducted in R version 3.6.1 (51). Data are available at https://doi.org/10.6084/m9.figshare.22680283 (58).

### Northward Range Shift and Phenotypic Flexibility in Migratory Destination.

**Fig. 5.**
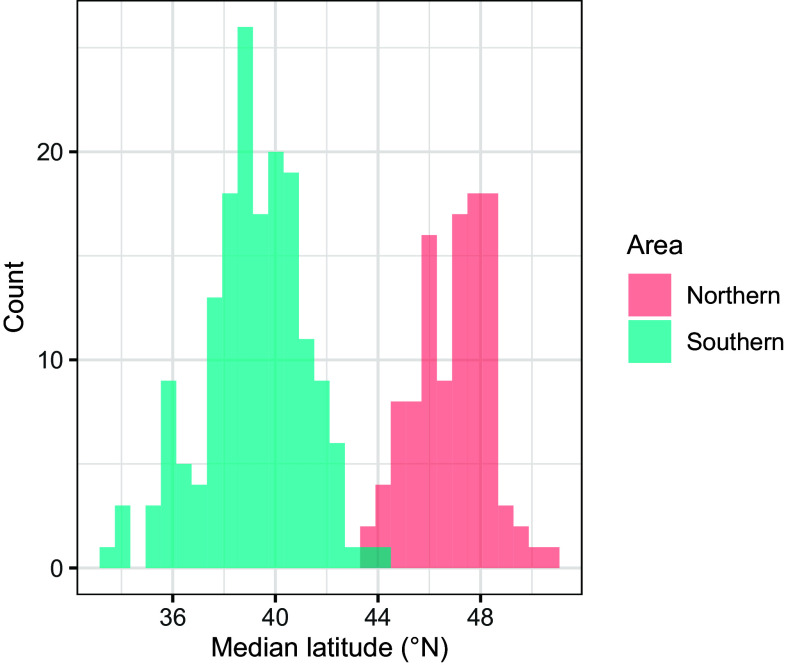
The distribution of median latitudes over all years of the study, colored according to whether they were identified as being part of the northern or southern area.

To summarize the distribution of Balearic shearwaters in the post-breeding season, we calculated each individual’s median latitude during migration. The distribution of median latitudes was highly bimodal, owing to the discrete areas occupied by the shearwaters around Iberia in the south and Brittany in the north. We used mixture models to identify whether a median latitude belonged to the northern or southern area. Given northward range shift, mixture models were not applied to the whole dataset. Conversely, single years provided too few tracks, with too incomplete a distribution to identify the two areas using mixture models in a repeatable way. Instead, tracks were grouped into three batches of three consecutive years (2010 to 2012, 2013 to 2015, and 2016 to 2018), and a mixture model was applied to the distribution of median latitudes in each batch. The results are seen in Fig. 5.

To test for range shift, we used a linear regression with median latitude as the response and year as the predictor, including each bird’s first track only, to avoid pseudoreplication and age-related changes in latitude. In this model, we also accounted for the two separate areas (north/south), sex (male/female), and island (Mallorca/Ibiza) (see Table 1 for full model structures).

**Table 1.** Full model structures showing the response variable, type of model, assumed distribution of residuals, fixed and random effects

Response	Model type	Distribution	Fixed effects	Random intercept
Median latitude	Linear regression	Normal	Year, area, sex, and island of origin	NA
Median latitude	Linear mixed model	Normal	Between-individual SST, within-individual SST, area, and sex	Individual
Log odds of migrated to the northern area	Generalized linear mixed model	Binomial	Between-individual SST, within-individual SST, area, island of origin, and sex	Individual
Return speed	Linear mixed model	Normal	Between-individual max. lat., within-individual max. lat., outbound speed, area, and sex	Individual
Departure date	Linear mixed model	Normal	Between-individual max. lat., within-individual max. lat., area, and island of origin	Individual
Return date	Linear mixed model	Normal	Between-individual max. lat., within-individual max. lat., area, and island of origin	Individual

To test whether this effect of year reflected climatic effects, we constructed an identical model but with mean SST as the predictor, instead of year. These models were compared to identify the best fitting model and thus determine whether SST, as a proxy for climate, better explained latitudinal shifts than time alone. Model comparisons were carried out using AICc, the small-sample corrected Akaike Information Criterion from the “AICcmodavg” package (59).

To investigate range shift further, we constructed linear mixed-effects models using the “lme4” package (60). For all mixed effects models, an individual was included as a random intercept. Significance was assessed with likelihood ratio tests on nested full and null models. Simulations were carried out using the “arm” package (61) to estimate bootstrapped 95% CI on effect sizes and to estimate the 95% CI of the difference between effect sizes (62). Full model structures are reported in Table 1 and full results are reported in *SI Appendix*, Table S1.

**Fig. 6.**
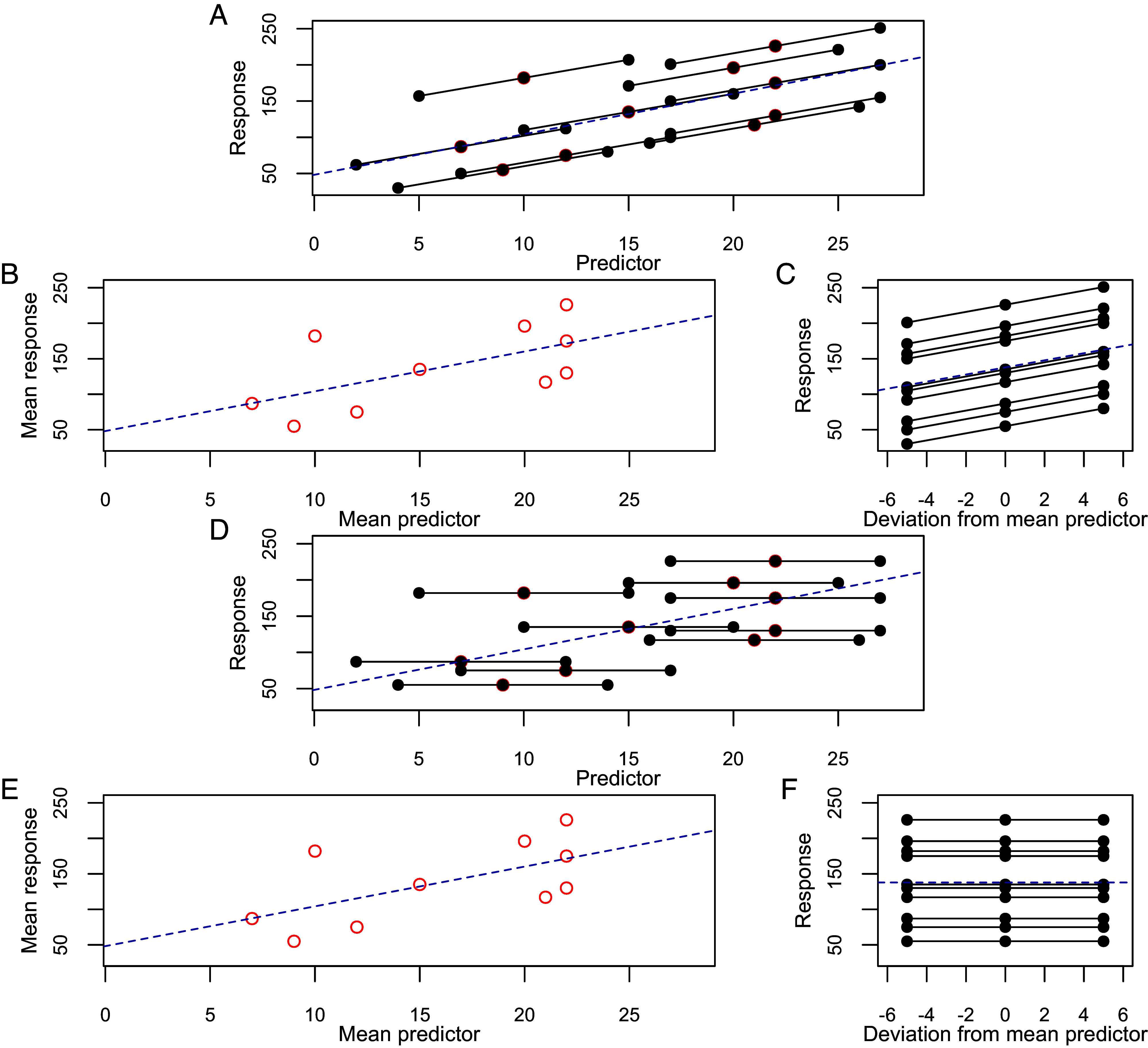
The subject centering method of van de Pol & Wright (36). This method allows us to distinguish whether changes in behavior observed at the population level are facilitated by individual flexibility or turnover of individuals with fixed strategies. (*A*) Hypothetical observations of a response to a predictor for 10 individuals, each with 3 observations. Each individual’s observations are shown with black points and joined by the black lines. The mean predictor and response for each individual are shown with a red circle. The overall relationship is shown with the blue dashed line. The black lines have a very similar slope to the overall relationship, indicating that the population-level change can be explained by flexible within-individual responses to the predictor. (*B*) The between-individual relationship can be estimated using the mean predictor and response for each individual; it is identical to the overall relationship. (*C*) The within-individual relationship can be estimated by expressing the predictor at each observation as a deviation from the mean predictor experienced by each individual. The relationship here is very similar to the overall relationship, indicating that the overall population-level change can be explained by within-individual flexibility. (*D*) Observations of a different response from 10 individuals, as in panel *A*. Here, the black lines have a gradient of 0, indicating that each individual has a fixed behavior and does not respond to the predictor flexibly. However, the overall population-level change is identical to *A*. (*E*) The relationship between individual’s mean predictors and responses is the same as in *B*. (*F*) By expressing the predictor at each observation relative to the mean predictor experienced by each individual, it can be seen that the within-individual slope is 0. As the between-individual slope is much greater than 0, the population-level change in this response is facilitated by turnover between individuals, each of which has a fixed behavior.

Model construction followed the method of van de Pol & Wright (36) to partition the relationship between median latitude and temperature into within-individual (phenotypic flexibility) and between-individual (turnover) effects, using subject-centering. For each individual, we calculated the mean temperature over all the years it was tracked. For each year it was tracked, we then calculated the deviation from this individual mean (Fig. 6). Differences between the mean temperatures experienced by different individuals express the variation in the predictor between individuals. Deviations from each individual’s within-subject mean express the variation in the predictor within an individual. The relationship between the between-individual means and the behavior of interest quantifies the population-level effect of the predictor, e.g., individuals in the study population experiencing different average temperatures have different average latitudes. The relationship between the within-individual variation and the behavior of interest quantifies the within-individual flexible response, e.g., when a single individual experiences different temperatures, it changes its migratory latitude. Therefore, a significant effect of the within-individual component indicates an at least partially flexible response. If the effect of the between-individual component is significantly different from the within-individual effect, this suggests that the population-level change is not fully explained by within-individual responses. In this case, there must be turnover of individuals with different average behaviors, in addition to any flexible response.

Given the two post-breeding areas occupied by Balearic shearwaters, the observed northward range shift could reflect birds a) changing destination from the southern to northern area, b) shifting in latitude within each area, or c) both. We, therefore, applied the same subject-centering method to test whether the probability of migrating to the northern or southern area was associated with temperature, using a generalized linear mixed effects model with a logit-link function. In this model, the northern area was scored as 1 and the southern area as 0 and island and sex were included as fixed effects.

### Differences in Return Speed Based on Route Memory.

To test whether daily speed during return migration was associated with maximum latitude, we used the latitude at the apex and calculated the average daily speed over all subsequent fixes before the bird first returned to the Mediterranean Sea. We then calculated the variation in maximum latitude both within- and between-individuals using the same subject-centering method from van de Pol & Wright (36) and constructed a linear mixed effects model with between-individual maximum latitude, within-individual maximum latitude and area as fixed effects. To control for physically constrained individual differences in speed and the possibility that faster birds inherently reach higher latitudes, we also included the mean outbound speed in our model, so that residual variation probably reflected decisions made by the bird.

We tested whether shearwaters were aware of their increased return distance and so increased their return speed from the start of migration. We then determined what kind of spatial representation they used to estimate this distance. We calculated the average daily speed over the first 25% period of each return migration and constructed three linear models, each with just one fixed predictor: beeline distance, route distance or maximum latitude. Both distances were calculated from the apex migration position to the colony. The beeline distance was calculated using the great circle distance using the Haversine function. The route distance was calculated as the shortest route from the apex fix to the colony without crossing over land. When defining the start of return migration using the latitudinal apex, there is a risk that the latitude identified will result from inaccurate geolocation which yields an extreme latitude estimate. Such erroneous estimates will inherently be associated with higher estimated speeds because, on average, they fall further from the preceding accurate location estimates than an accurate estimate would. We included the model using maximum latitude for this reason—if the speed-distance relationship were driven by the inherent link between erroneous latitudinal geolocation and estimated speed, latitude should be a better predictor of initial speed than either distance metric. We also constructed a model to rule out that differences in return speed were simply the result of a confound between maximum latitude and the area/habitat crossed during migration. To do this, we calculated average daily speed over a fixed latitudinal band (35°N to 45°N) where all shearwaters must be migrating over the same area.

### Phenological Shifts.

To test for changes in phenology associated with changing migratory latitudes, we constructed two linear mixed effects models, one with departure date from the Mediterranean Sea and one with return date. Both models included within- and between-individual variation in maximum latitude, as well as area and island as fixed effects.

## Supplementary Material

Appendix 01 (PDF)

## Data Availability

Tracking data have been deposited in FigShare (https://doi.org/10.6084/m9.figshare.22680283) (58).
