# Supplementary material for: Climate change drives migratory range shift via individual plasticity in shearwaters
Source: Proc Natl Acad Sci U S A. 2024 Jan 29;121(6):e2312438121. doi: 10.1073/pnas.2312438121 (PMC10861922; doi:10.1073/pnas.2312438121)
Supplement: Supplementary file 1 — Appendix 01 (PDF) [file pnas.2312438121.sapp.pdf]

PNAS

Supporting Information for

Climate change drives migratory range shift via individual plasticity in shearwaters.

Patrick J. Lewin*, Joe Wynn*, José Manuel Arcos, Rhiannon E. Austin, Josephine Blagrove, Sarah Bond, Gemma Carrasco, Karine Delord, Lewis Fisher-Reeves, David García, Natasha Gillies, Tim Guilford*, Isobel Hawkins, Paris Jaggers, Christian Kirk, Maite Louzao, Lou Maurice, Miguel McMinn, Thierry Micol, Joe Morford, Greg Morgan, Jason Moss, Elisa Miquel Riera, Ana Rodriguez, Katrina Siddiqi-Davies, Henri Weimerskirch, Russell B. Wynn and Oliver Padget*.

*Patrick Lewin, Joe Wynn, Oliver Padget, Tim Guilford
Email: patrick.lewin@biology.ox.ac.uk, joseph.wynn@ifv-vogelwarte.de, oliver.padget@liverpool.ac.uk, tim.guilford@biology.ox.ac.uk

This PDF file includes:

Supporting text
Figures S1 to S3
Table S1

Supporting Information Text

Geolocator processing and identification of migration

The buffer designed to account for geolocator error when identifying crossings through the Strait of Gibraltar, in or out of the Mediterranean Sea, ran between 6.6°W 3°0N, 6.6°W 45°N, 1°W 45°N, 1°W 60°N, 90°E 60°N, 90°E 43°N, 1°W 43°N, 1°W 40°N, 4.6°W 40°N and 4.6°W 30°N (Fig. S1). Crossings of this buffer, indicating the initiation or end of migration, were identified when the a rolling mean longitude and latitude position over a window of three fixes crossed from one side of the buffer to the other, as shown in Fig.S2.

Survival and breeding success

To assess whether climatic changes in the post-breeding area and associated changes in migratory behaviour were affecting the demographic rates of Balearic shearwaters, we conducted a survival analysis of tracked birds and an analysis of breeding success at the population and individual levels.

To conduct a survival analysis, we made the assumption that the probability of geolocator retrieval (and so continued presence in the study population) was correlated with the probability of survival. The entry and disappearance of birds from the geolocator tracking is shown in Fig.S3. We implemented a Cox proportional hazard model using the ’coxph’ function from the ’survival’ package in R. The predictor was each bird’s median latitude in the first year it was tracked, relative to the population average in that year, and the response was the number of years from the bird’s first track to its last, censored at the end of the study in 2018. We found that relative median latitude did not predict longevity in the study: the estimated hazard ratio was 0.999 (n = 145, SE = 0.028, p = 0.69).

Our analysis of breeding success involved data from Mallorca between 2011 and 2015. At the population level, we analysed the probability of egg laying in annually monitored study nests. Among those nests in which a chick hatched, we also conducted an analysis of the probability of fledging. Both outcomes were analysed in relation to the mean July and August SST in the post-breeding area in the previous year using a generalised linear model with a logit link. There was no effect of climate in the migratory area on either of these outcomes (egg-laying: n = 406, log odds change = 0.252 per °C, SE = 0.249, p = 0.311; fledging: n = 185, log odds change = 0.672 per °C, SE = 0.600, p = 0.263). Since geolocators were only retrieved from active breeders, it was not possible to analyse the relationship between individual migratory behaviour and egg-laying. However, we were able to investigate fledging success at the individual level in relation to the individuals’ migratory latitudes in the previous migration. We again used a generalised linear model with a logit link and found that migratory behaviour did not significantly affect subsequent fledging success (n = 39, log odds change = 0.202 per °N, SE = 0.264, p = 0.445).

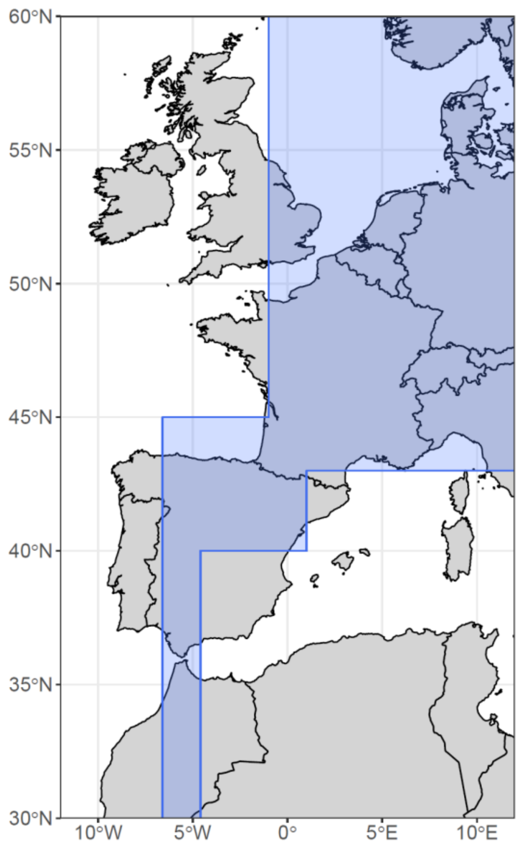

Fig. S1. The buffer area used to mitigate against geolocator error when identify crossings from the Mediterranean Sea to the Atlantic Ocean.

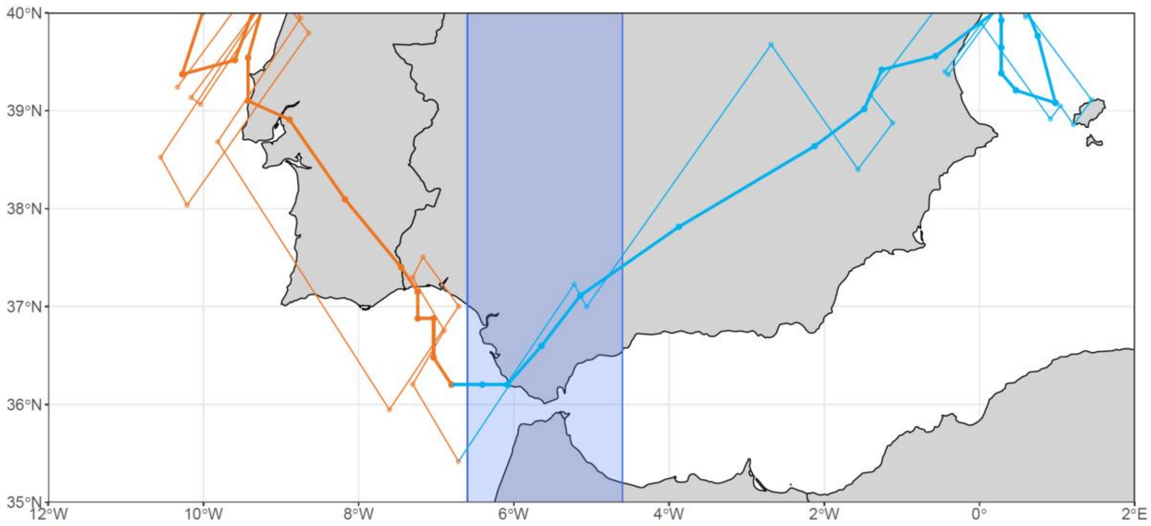

Fig. S2. An example of a single crossing from the Mediterranean Sea (fixes shown in blue), over the buffer area, to the Atlantic Ocean (fixes shown in orange). The thinner lines and points are raw location estimates, the thicker lines and points are the smoothed positions used to identify crossings and reduce the effect of geolocator error.

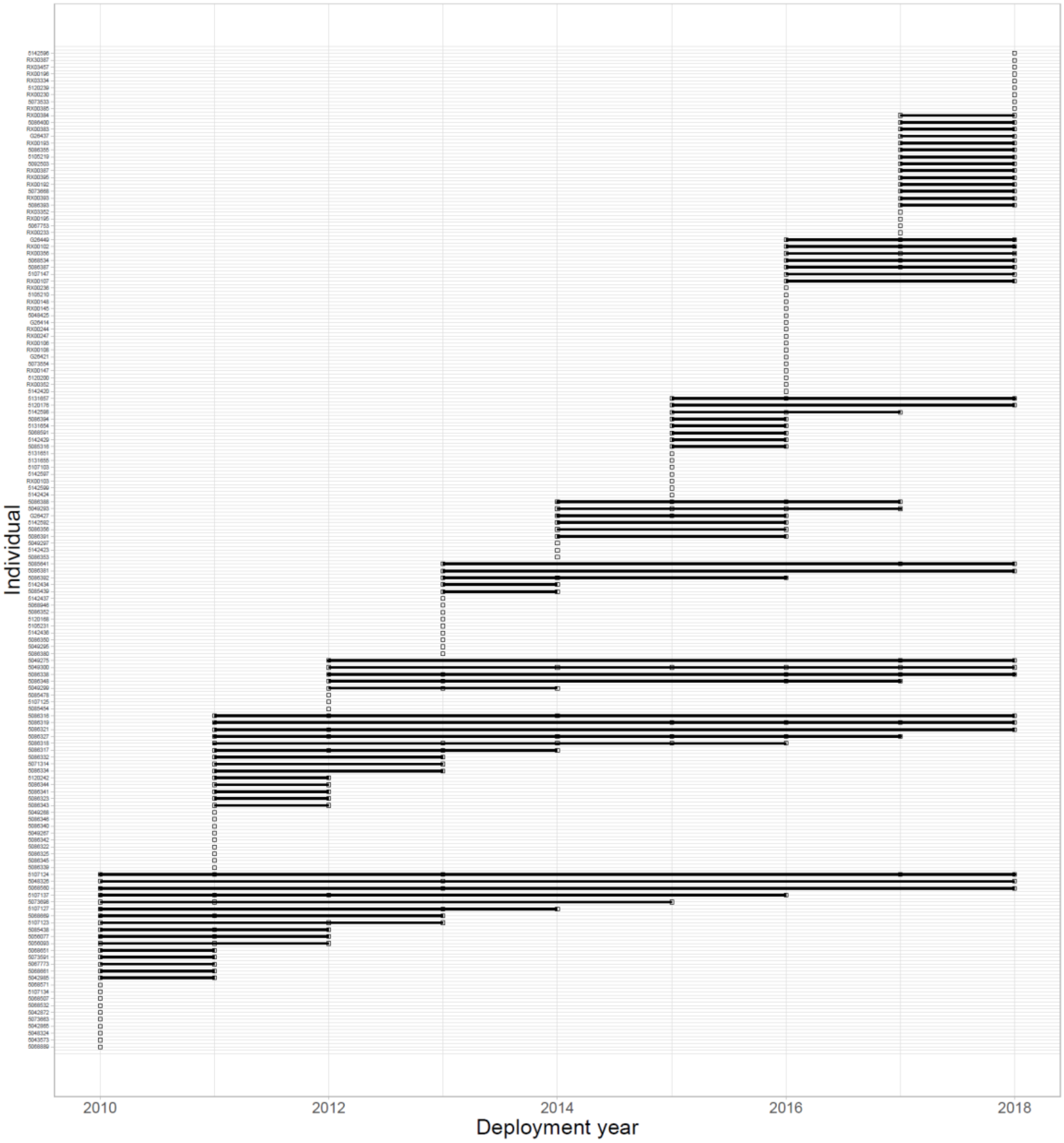

Fig. S3. A visualisation of turnover in the study population - individuals are arranged along the y-axis and points represent years in which they are tracked. The points, from the year each bird was first tracked to the year each bird was last tracked, are connected by horizontal black lines.

Table S1. Full results for all mixed effects models. The confidence intervals were estimated using bootstrapping and the chi-squared and p-values were estimated from likelihood ratio tests on full and null models for each term. Significant predictors are highlighted in bold and the level of significance is indicated by asterisks.

Response	Coefficient	Effect size	Confidence intervals	Chi-squared	p-value
Median latitude	Intercept	20.070	5.886, 34.956	-	-
	**Between-individual SST**	1.424	0.638, 2.272	12.119	<0.001***
	**Within-individual SST**	0.663	0.018, 1.331	4.050	0.044*
	**Area** (southern)	-7.177	−7.786, −6.564	225.039	$<1x10^{-15}$***
	**Sex** (male)	−0.677	−1.244, −0.109	5.041	0.025*
	Random intercept (individual)	0.482	0.348, 0.650	-	-
Area (log odds of migrating to northern area)	Intercept	-0.891	-4.934, 2.852	-	-
	**Between-individual SST**	1.791	−0.234, 3.893	4.560	0.033*
	Within-individual SST	0.004	−0.534, 0.579	0.000	0.988
	Island (Mallorca)	2.014	−2.414, 6.678	1.246	0.264
	**Sex** (male)	−6.882	−11.734, −2.197	6.181	0.013*
	Random intercept (individual)	39.640	28.288, 51.744	-	-
Return speed	Intercept	2.092	−6.304, 10.343	-	-
	**Between-individual max. lat.**	0.223	0.063, 0.388	8.116	0.004**
	**Within-individual max. lat.**	0.218	0.015, 0.413	4.541	0.033*
	Outbound speed	0.117	−0.026, 0.263	2.501	0.114
	Area (southern)	0.959	−0.216, 2.215	2.634	0.105
	Random intercept (individual)	1.162	0.853, 1.542	-	-
Departure date	Intercept	131.053	77.877, 174.301	-	-
	Between-individual max. lat.	0.697	−0.182, 1.734	1.983	0.159
	Within-individual max. lat.	0.267	−0.912, 1.456	0.189	0.664
	**Area** (southern)	11.656	4.102, 18.631	9.608	0.002**
	Island (Mallorca)	1.415	−4.972, 7.623	0.195	0.659
	Random intercept (individual)	92.400	68.812, 117.185	-	-
Return date	Intercept	−3.010	−52.083, 45.583	-	-
	**Between-individual max. lat.**	5.368	4.385, 6.279	82.092	$<1x10^{-15}$***
	**Within-individual max. lat.**	4.349	3.179, 5.616	41.499	$<1x10^{-10}$***
	**Area** (southern)	38.747	31.730, 46.759	81.438	$<1x10^{-15}$***
	**Island** (Mallorca)	−9.791	−16.994, −3.666	8.523	0.004**
	Random intercept (individual)	87.214	66.432, 111.299	-	-

Significance Levels: * p < 0.05, ** p < 0.01, *** p < 0.001
